# Paying Attention to Memory

**DOI:** 10.1371/journal.pbio.0020407

**Published:** 2004-10-26

If you could peer inside someone else's head, you'd see a scrunched-up gelatinous mass of tissue, weighing roughly a kilogram, homogeneous to the naked eye—in other words, a brain. The seeming uniformity of the overlying cerebral cortex, which has so outstripped other parts of the brain over the course of evolution that it makes up more than 80% of the brain, is belied by centuries of painstaking neuroscience. Some of the most compelling early evidence that parts of the cortex are specialized in their duties came from gun-shot wounds during the first world war. For instance, bullets lodged in the back of the brain disrupted sight in discrete portions of the visual scene, prompting insights into the localization and function of visual cortex.

The study of the front of the brain has a similar history of injury leading to insight. Phineas Gage, a railroad worker, had a 3.5-foot-long tamping iron blow straight through his frontal lobes and turned from a responsible, mild-mannered geek into an unruly exhibitionist overnight. Parts of the prefrontal cortex that he damaged have since been much studied for their involvement in motivation and emotional control.

More recent work has implicated other parts of the prefrontal cortex in working memory. Working memory is famously illustrated by your ability to temporarily remember a seven-digit telephone number, roughly the amount of information that you can store on-line in working memory for the duration of a task like phoning for a pizza.

Monkeys can be trained to remember information much like you remember a phone number, and then use the memory for gaining a reward (usually juice rather than pizza). They can learn to remember the specific location of a briefly flashed target on a screen and then, when cued, make an eye movement to look directly at that location. Previous research has shown that neurons in the prefrontal cortex maintain high rates of activity while monkeys remember the target location, and gradually the idea that the prefrontal cortex specializes in maintaining these transient memories has risen to dominance over other ideas about its functions.

In this issue of *PLoS Biology*, Mikhail Lebedev and his colleagues challenge this prevailing view with evidence that most prefrontal cortex neurons may not be so closely tied to working memory after all. As in previous research, they also trained monkeys to make an eye movement to a remembered target, but instead of only seeing one target, the monkeys saw two potential target locations during the course of the task. The monkeys had to pay attention to one of the potential targets, but this was not necessarily the one they would have responded to and was not the one they had to remember. To perform the task successfully, the animals had to engage their working memory, but most of the neurons the researchers recorded increased their activity selectively to the target that was the focus of attention.

Despite decades of research, the degree to which one region of the brain can be thought of as dedicated exclusively to a particular function is still much debated. These results do not refute the idea that the prefrontal cortex plays an important role in working memory. However, the authors suggest that this area may be more important in focusing the attention needed to remember that phone number, rather than actually holding that number in your mind.

